# Salvage of a severed Paul Glaucoma Implant tube via anastomosis with an Ahmed Glaucoma Valve in a child with Sturge–Weber syndrome and complicated glaucoma: a case report

**DOI:** 10.1097/RC9.0000000000000536

**Published:** 2026-05-20

**Authors:** Dania Bamefleh, Mohammed Halawani, Hajar Alotaibi, Alnuad AlBazei, Anas Alqurashi

**Affiliations:** a Glaucoma Division, King Khaled Eye Specialist Hospital & Research Center, Riyadh, Saudi Arabia; b Faculty of Medicine, Umm AlQura University, Makkah, Saudi Arabia

**Keywords:** Ahmed Glaucoma Valve, Paul Glaucoma Implant, salvage implant, Sturge–Weber syndrome, tube anastomosis

## Abstract

**Introduction:**

Refractory glaucoma in Sturge–Weber syndrome (SWS) is challenging to manage, with a high risk of intraoperative complications. The Paul Glaucoma Implant (PGI) is a novel device with smaller inner and outer tube diameters than the Ahmed Glaucoma Valve (AGV).

**Importance:**

To describe a creative intraoperative salvage technique for a transected PGI, using a hybrid tube anastomosis with a segment of AGV tube, in a pediatric patient with SWS.

**Case presentation:**

A 5-year-old girl with SWS and uncontrolled glaucoma underwent revision surgery 5 months after PGI implantation. Intraoperatively, the PGI tube was inadvertently transected. Rather than replacing the entire implant, which could lead to unwanted intraoperative and postoperative hypotony as well as promote additional fibrosis of the bleb, a segment of AGV tube (~6 mm) was anastomosed to the remaining PGI tube, creating a hybrid conduit. Postoperatively, the patient remained stable without complications over a 6-month follow-up.

**Clinical discussion:**

The AGV tube can be expanded to receive the smaller PGI tube, making a watertight anastomosis. Compared to the commercial tube extender, this method is less bulky and therefore has a lower risk of exposure and erosion. This method may help reduce surgery time, especially in high-risk patients like those with SWS, where implanting another PGI in a different quadrant or explanting the device for another surgery could cause complications related to the syndrome.

**Conclusion:**

This hybrid tube approach, combining PGI and AGV components, may represent a feasible method for salvaging damaged glaucoma drainage devices in critical intraoperative settings. However, longer-term observation is needed to better evaluate this technique.

## Introduction

Refractory glaucoma is one of the most common and challenging ocular manifestations in patients with Sturge–Weber syndrome (SWS), a rare congenital neuro-oculocutaneous disorder, occurring in up to 70% of patients with SWS^[^1–5^]^. There is no standard approach to managing SWS glaucoma, as most patients are resistant to medical treatment and have low success rates with laser and traditional filtering surgeries due to intraoperative and post-surgical complications^[^3–5^]^. Glaucoma drainage devices (GDDs), such as the Ahmed Glaucoma Valve (AGV), have been reported to be highly effective in controlling intraocular pressure (IOP) in refractory glaucoma associated with SWS, and with fewer complications^[^4^]^. The Paul Glaucoma Implant (PGI) is a newer non-valved GDD made of biocompatible medical-grade silicone, featuring a smaller inner tube diameter of 0.127 millimeters (mm) and an external diameter of 0.467 mm compared with the AGV (0.305 mm internal lumen diameter and 0.630 mm external diameter, respectively)^[^6–8^]^. When GDDs are compromised by a tube defect, a few studies have described replacing only the tube part while preserving the functioning plate, such as tube-extension or tube-in-tube techniques during planned revision surgery rather than incidental intraoperative tube transection in a high-risk child^[^9^]^. The current study reports a rare incident of intraoperative transection of the PGI tube during revision surgery and describes a surgical technique for salvaging the implant by creating a hybrid tube through anastomosis with an AGV upper segment tube. This case report has been prepared in accordance with the SCARE 2025 criteria^[^10^]^.

HIGHLIGHTS

- Creative intraoperative salvage technique for a transected Paul Glaucoma Implant.
- Replacing the implant can lead to intraoperative and postoperative complications.
- The anastomosis site showed no leakage with a 30-gauge balanced salt solution.
- After 6 months, the patient’s intraocular pressure was stable without complications.
- Hybrid tube approach is a safe and effective method in critical intraoperative settings.

## Patient information

A 5-year-old girl, with known SWS involving the right side of her face and encephalotrigeminal angiomatosis, presented to King Khaled Eye Specialist Hospital and Research Center with ocular manifestations of refractory glaucoma in her right eye. She had a history of seizure disorder and had undergone a hemispherectomy, but at presentation, she had been off medication for 3 years with no recent attacks. The child also had left hemiparesis, predominantly affecting the upper arm, which was gradually improving with physiotherapy. Her past ocular history included three glaucoma interventions for the right eye. First, she underwent deep sclerectomy with mitomycin-C, followed by micropulse cyclophotocoagulation, and subsequently a PGI with intraluminal 7-0 Prolene ripcord due to uncontrolled IOP. However, 4 months post-PGI implantation, the IOP remained above the target range, reaching 40 mm of mercury (mmHg), despite medical and surgical interventions. The child was receiving dorzolamide/timolol twice daily and latanoprost every night at bedtime. On examination of the right eye, the central corneal thickness was 602 μm, with buphthalmos, a clear cornea, a deep anterior chamber (AC), and the PGI tube touching the corneal endothelium. Fundus examination showed a choroidal hemangioma with a cup-to-disc (C/D) ratio of 0.8. External examination revealed a port-wine stain involving the ophthalmic, maxillary, and mandibular divisions of the trigeminal nerve. The axial length was 26.28 mm. The left eye had an IOP of 22 mmHg, and the C/D ratio was 0.4. Given the persistently elevated IOP in the right eye, the decision was made to proceed with revision surgery to remove the ripcord and reassess the position of the PGI.

## Surgical technique

**Figure 1.**
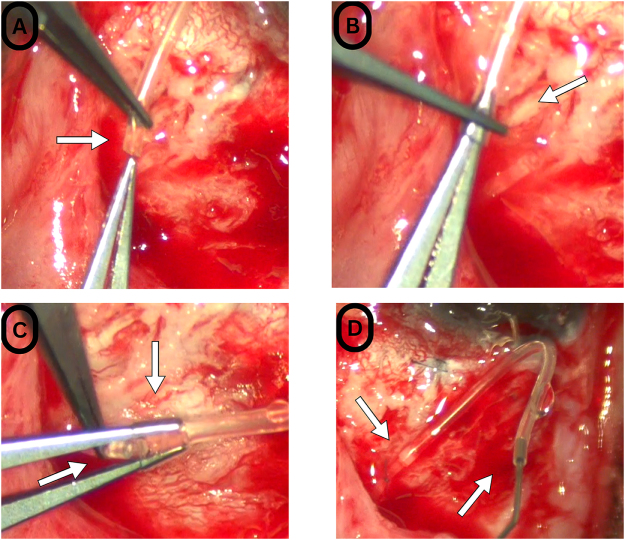
Intraoperative images. (A) The Ahmed Glaucoma Valve (AGV) tube (white arrow) stabilized at its orifice with tying forceps to introduce the closed tips of the Madison forceps into the AGV lumen. (B) The Madison forceps are advancing into the AGV lumen at the maximum distance (~2 mm) while the tying forceps are holding the tube from the tip. (C) The AGV lumen was widened via the Madison forceps (upper white arrow) in preparation for slipping the Paul Glaucoma Implant (PGI), which is held via tying forceps, into the AGV (lower white arrow). (D) PGI–AGV anastomosis site (upper white arrow) was tested for leakage using the balanced salt solution irrigation through a 30-gauge cannula (lower white arrow).

The procedure was performed at a tertiary eye care referral hospital and research center by a senior glaucoma surgeon experienced in glaucoma surgical procedures. The operative field was sterilized in the standard manner under general anesthesia. A wire speculum was inserted between the eyelids, and a corneal traction suture was placed using 7-0 Vicryl. A fornix-based conjunctival peritomy was performed in the superotemporal area, which was extensively fibrosed and congested with multiple tortuous vessels – a finding commonly seen in patients with SWS, which may be related to elevated episcleral venous pressure. The previously placed pericardial patch was retracted to evaluate the tube and remove the ripcord, which was positioned behind the patch. While liberating the tube from the fibrosis, the PGI tube was accidentally transected posteriorly. This highlights the importance of adequate visualization and careful identification of instrument position before tissue cutting. The decision was made to salvage the PGI rather than explant and replace it, which could lead to unwanted intraoperative and postoperative hypotony, as well as promote additional fibrosis of the bleb, resulting in worse long-term function, especially for a pediatric patient with SWS. A new AGV was unpacked, and a tube segment was cut to approximately 6 mm in length and separated from the plate. The AGV tube was stabilized at its orifice with tying forceps, and the closed tips of Madison forceps were then introduced into the AGV lumen for a maximum distance of approximately 2 mm. The PGI tube was held at its tip with tying forceps and advanced approximately 2 mm into the AGV lumen while the Madison forceps stretched the tube. The junction integrity was assessed by irrigating with a 30-gauge balanced salt solution, and no leakage was observed (Fig. 1). The ripcord was removed. The tube was ligated using 8-0 Vicryl posterior to the anastomosis to avoid postoperative hypotony, which in SWS poses significantly elevated risks of choroidal hemorrhage and severe choroidal detachment – risks that are further increased with an axial length of 26.28 mm. The previous sclerotomy was closed using 10-0 nylon, and a new sclerotomy was created to insert the new hybrid tube using a 23-gauge needle. The hybrid tube was trimmed to an appropriate length, then inserted into the AC and secured to the sclera using 10-0 nylon. It was then covered with a new pericardial patch graft and secured in place with 7-0 Vicryl. Finally, the conjunctiva was securely closed with a continuous 9-0 Vicryl suture. A mixture of 2 mg dexamethasone and 50 mg/0.5 mL of cefazolin was administered in the subconjunctival space using a 30-gauge needle. The lid speculum was removed, and a double patch and eye shield were placed for protection.

## Outcomes

On the first postoperative day, the patient was stable, with IOP reduced to 18 mmHg in the right eye owing to the ripcord removal. Six months after the procedure, IOP was measured at 12 and 14 mmHg in the right and left eyes, respectively. The right eye remained controlled on brinzolamide/timolol twice daily. The tube remained in a stable position, and the AC was deep. No postoperative complications were observed.

## Discussion

Tube transection, a too-short trimmed tube, a retracted tube, and an obstructed tube are examples of complications that may occur intraoperatively during revision surgery or while implanting a GDD, which can benefit from extending the tube to preserve or avoid explantation of the GDD.

The first method for lengthening the tube was reported by Kooner *et al*, using a Silastic extension tube that connects the two ends of the Molteno implant tube and is secured to the sclera at three points with 10-0 nylon sutures^[^11^]^. Other materials have been suggested to lengthen the tube, including an angiocatheter, a 22-gauge intravenous catheter, a Crawford tube, a XEN stent, and a Preserflo MicroShunt^[^12–16^]^. However, some of these materials are not well-studied for long-term intraocular biocompatibility, with unpredictable long-term effects. Therefore, using a tube from another GDD may carry a lower risk, although it may be more expensive. Additionally, using micro-invasive glaucoma surgery tubes as an extension to GDDs could increase the risk of blocking the junction, and the outcome could be affected due to the much smaller tube size and diameter^[^9^]^. The commercially available tube extender (Model TE; New World Medical) might be used in our case, but it is too bulky, with no evidence for pediatric eyes, and this carries the risk of erosion and exposure, especially with scarred and friable conjunctiva from previous procedures^[^17^]^.

Alternative management for the child may include implanting another GDD in another quadrant while preserving the PGI plate. This will result in a longer operating duration, additional inflammation, and bleeding. Moreover, exchanging the plate with another GDD may risk compromising the bleb, which would result in undesirable hypotony with a high risk of complications or failure. Furthermore, SWS patients have a high risk of sudden IOP shifts, which may result in choroidal effusion and even hemorrhage^[^18^]^. The tube-in-tube techniques are not risk-free; fibrosis, erosion, or occlusion theoretically may occur at the anastomosis site, and long-term efficacy and safety have not been reported.

A few studies have reported using another tube from a new GDD as an extension^[^9,15,19,20^]^. Gillmann *et al* reported using the same type of GDD (Baerveldt implant) to salvage a tube that was blocked by fibrin, with the two tubes then stitched together using two 8-0 nylon^[^20^]^. More recently, Berman *et al* (2024) used a PGI tube to extend an implanted AGV and Baerveldt implant without employing any suturing^[^9^]^. In the present report, we describe a variation of Berman’s procedure in reverse configuration, where an AGV tube segment was used to lengthen the transected PGI tube. Because the AGV tube’s internal lumen (0.305 mm) is large enough, when stretched, to accommodate the PGI tube’s outer diameter (0.467 mm), the anastomosis site is secured by a pressure-fit junction alone, making suturing unnecessary. The trade-off for losing the smaller PGI lumen may introduce long-term complications, such as a higher risk of hypotony, especially after removing the ripcord, and a higher rate of corneal endothelial cell loss (ECL). A prospective study reported mean central ECL losses of 15.3% at 12 months and 18.6% at 24 months^[^21^]^. Compared to PGI, a systematic review reports low rates of corneal decompensation (4.1%), but head-to-head comparisons have not yet been conducted^[^22^]^. Other late complications may include tube occlusion, fibrosis, or erosion at the anastomotic junction. Therefore, although the present technique appears to be a feasible rescue approach in selected scenarios, its long-term safety and durability should be carefully considered.

The current study reports a creative method to salvage PGI using a hybrid tube made of PGI–AGV after intraoperative tube transection during the operation. Longer-term studies are needed to evaluate efficacy and potential complications, such as occlusion, fibrosis, and erosion at the anastomotic site, which is challenging because this technique is considered only in selected scenarios.

## Conclusion

The hybrid tube anastomosis, achieved by combining PGI and AGV tubes, is a novel and simple surgical technique for managing complications such as tube transection during a tube revision or after scleral fixation, especially in scenarios where preserving the existing GDD is essential for controlling the IOP and where opening the bleb to manipulate or remove the plate would jeopardize the capsule and cause severe hypotony, as seen in the present case. However, given the limited follow-up period of 6 months, longer-term observation is necessary to better evaluate the long-term safety and effectiveness of this technique.

## Data Availability

All data relevant to this case report are included in the article.
